# Initial Design of a Self-Retracting Trocar for Distal Ventriculoperitoneal Catheter Insertion

**DOI:** 10.7759/cureus.102065

**Published:** 2026-01-22

**Authors:** Jacques Lara-Reyna, Grant Ganpat, Michael Larson, Caleb Lee, Brendan Mason, Kalyani Nair, Julian J Lin

**Affiliations:** 1 Neurosurgery, University of Illinois College of Medicine at Peoria, Peoria, USA; 2 Mechanical Engineering, Bradley University, Peoria, USA

**Keywords:** cerebrospinal fluid, hydrocephalus, peritoneum, shunt, trocar

## Abstract

Background

Laparoscopic access, assisted by a general surgeon, is the most commonly used technique for inserting a distal catheter during ventriculoperitoneal shunt placement. This study introduces a novel single-use trocar designed for peritoneal access, combining engineering analyses to ensure performance and safety.

Methods

We presented the design and bench evaluation of this new trocar concept. A Pugh matrix evaluated material and feature optimization, while failure mode and effect analysis (FMEA) identified risks such as blade dulling and spring failure. Finite element analysis (FEA) confirmed the structural integrity of the trocar.

Results

The FEA confirmed the structural integrity of the trocar, demonstrating high load capacities of 1655.3 N for the shaft, 503.4 N for the cannula, and 2799.12 N for the handle with minimal deformation and a high safety margin. The prototype features a sharp blade with an automatically retractable blunt tip to reduce tissue trauma and a cannula with a side slit for catheter insertion. Using sausage casing to simulate the peritoneum, sharpness testing showed a breakthrough force of 2.98 N compared to 2 N for the Versastep trocar (Medtronic, Minneapolis, MN).

Conclusions

The assessment and conceptualization of this new trocar showed reliability, a low risk of component failure, and good sharpness performance after multiple laboratory tests. The self-retractable tip provides a safety profile during insertion into the peritoneal cavity. Further testing in vivo is needed.

## Introduction

Hydrocephalus is the abnormal accumulation of cerebrospinal fluid (CSF) with subsequent dilation of the ventricular system. Different etiologies can lead to the development of hydrocephalus, which can affect patients of various ages [1].

Hydrocephalus is most commonly treated by inserting a shunt to divert the flow of CSF from the ventricles to a different area of the body, often into the peritoneal cavity [2]. Accessing the peritoneal cavity is usually done by mini-laparotomy. In recent years, a laparoscopic approach to distal catheter insertion has become more popular due to benefits such as lower rates of distal catheter failure, decreased blood loss, and shorter operative times [2]. However, this approach requires the assistance of a general surgery team. 

This project aims to present the design and bench evaluation of a new type of trocar for inserting the distal catheter that may avoid the need for laparotomy or endoscopic assistance during the catheter placement.

## Materials and methods

The following project involved creating a novel trocar for peritoneal access during the placement of distal catheters. The Department of Mechanical Engineering at Bradley University, Peoria, IL, completed the initial steps from conception to the final prototype.

The risk of intra-abdominal injury, the need for cannula removal, and the versatility of use in both pediatric and adult patients are some of the issues commonly encountered when using traditional trocars. Multiple port sites are also often necessary in traditional laparoscopic approaches, further increasing the risk of patient injury. We sought to address these issues by developing a single-operator, single-use device to increase surgeon efficiency and patient safety by eliminating the need for abdominal insufflation and multiple port sites. 

After initial conceptualization and solution ideation, the overall prototype was to have three main components: the inner trocar, the outer cannula, and a static measuring device on the cannula. 

The initial desired specification of the system involved a retractable blade at the tip of the trocar to aid in the removal of the device. While removing the cannula, the surgeon uses the attached measuring device to ensure the distal catheter remains inside the abdomen at the correct distance.

Quality function deployment (QFD) testing revealed that the primary desired characteristics of the device were related to hardware, precision, manufacturing quality, and device operation. Features such as trocar diameter, material strength, and the force required to penetrate tissue layers were also noted. 

A Pugh matrix was developed to determine which trocar design concept aligned most closely with the QFD-determined specifications. After selection, the chosen design was evaluated using a failure mode and effect analysis (FMEA) in which each element of the system underwent a finite element analysis (FEA) to test for structural integrity under maximum load and maximum deformation conditions [3,4].

## Results

The final report, after conceptualization, design, and prototyping, was completed in April 2024 and found that the most essential features of the trocar fell under the categories of “safety” and “ease of use.” The QFD results revealed that the most essential features of the trocar were sharpness and retractability of the blade and the overall ease and safety of the device during initial insertion. Individual assessments of the trocar system are as follows.

Trocar design

The final design is a sharp, retractable, stainless-steel blade within a blunt-tipped cannula. Once pressure is applied, the blade is exposed to cut through tissue layers. When pressure is released, the blunt tip protects the internal organs from damage, making this design safer than the base concept. A spring under the handle at the top of the trocar allows the blade to retract to maximum capacity. 

Cannula design

A solid reusable, stainless steel cannula with a slit in the side, allowing the shunt catheter to remain inside the peritoneum at the desired length during device removal, was deemed to be the most beneficial design when compared to other options (disposable cuttable, slidable or removable side door, or telescoping cannula).

**Figure 1:**
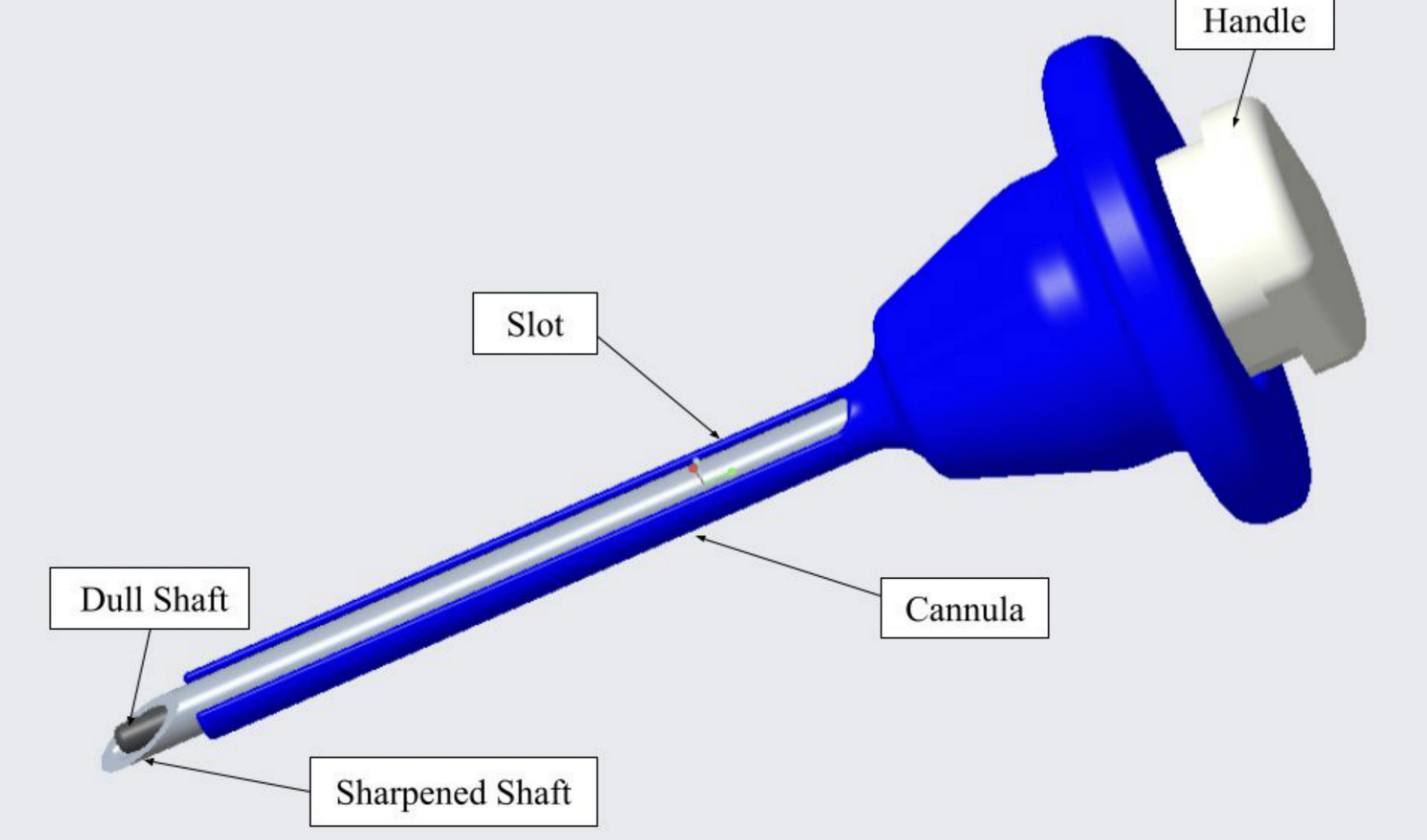
Trocar system with the dull shaft (dark gray) inside the retractable sharp blade shaft (light gray), cannula with a slit on the side (blue), and the system’s handle (white) Image credits: authors of this article.

The final dimensions of the selected prototype are listed in Table 1, and the prototype rendering is shown in Figure 1.

**Table 1:** Final proposed diameters of different elements

System element	Diameter (mm)
Sharp shaft outer diameter	4
Sharp shaft inner diameter	3
Cannula outer diameter	8
Cannula inner diameter	4.5
Handle inner shaft diameter	4.5

FMEA

After creating the trocar system designs, possible points of failure were evaluated using FMEA. An FMEA chart breaks down each part of a device and how it can fail. It then assigns the effects, causes, and detection techniques to determine how vital a failure is to design around and fix.

Trocar FMEA

Upon analysis, several potential trocar failure points were identified. Dulling of the blade is possible due to overuse as well as inadequate sharpening practices, impacting the ability of the blade to adequately penetrate tissue layers upon entry. Potential failure of the spring mechanism, which controls the deployment and retraction of the interior blade, may result in two possible outcomes. First, the blade may become stuck inside the cannula, unable to be deployed, rendering the device unusable. Conversely, the blade could potentially become stuck in the deployed position outside of the cannula, creating a patient safety risk. The final risk factor identified involves the potential of bending or fracturing of the trocar shaft during a procedure, rendering the device unsafe or unusable. 

Cannula FMEA

Several potential failure points were also identified in the cannula portion of the device. Chipping on the surface of the cannula may cause unintended cuts or abrasions to the patient’s skin during insertion and removal. Similarly, sharp edges on the open slot of the cannula may also cause damage to the patient’s skin. Finally, bending or breakage of the cannula through the trocar shaft could render the device unusable.

FEA

To test the structural integrity of the trocar system, each part of the system was tested for the maximum load (von Mises stress results), maximum deformation, and maximum strain under normal loading force. A factor of safety (FOS) was set at a minimum of 4. After researching this, all parts were analyzed with an expected work loading of 5 N as the maximum force required to puncture different tissue layers. A maximum deformation limit was set at 0.50% of the total length of the model.

Independent values for the sharp trocar shaft, the cannula, and the handle are seen in Table 2.

**Table 2:** Maximum load, deformation, strain, and FOS for different components of the system FOS, factor of safety

System component	Maximal load (N)	Maximal deformation (mm)	Maximal strain	FOS (minimum of 4)
Sharp trocar shaft	1655.3	0.00107	0.00014	80
Cannula	503.4	0.513	0.004	38
Handle	2799.12	0.0000361	0.0000009	300

From the FEA, it was determined that no parts would fail based on the loads and constraints applied.

Testing of the final prototype

After the FMEA and FEA demonstrated a low risk of failure of the elements, the final prototype was tested. A VersaStep trocar (Medtronic, Minneapolis, MN) was used for comparison.

Spring testing

A spring was placed into the trocar. The original distance between the tip of the blade and the tip of the blunt cannula was measured to determine displacement. The selected spring was placed into the trocar system, and the trocar was placed upside down on a zeroed scale with the units set to grams. A wooden block was used to apply force steadily to the tip of the trocar. The block was pushed until the sharp blade barely touched it, and the mass value was recorded.

Data was collected for the Versastep trocar and three compatible springs. The average spring factor for the Versastep trocar was about 804 N/m. After testing three different springs, a spring factor of 265 N/m was selected. 

Blade sharpness testing

**Figure 2:**
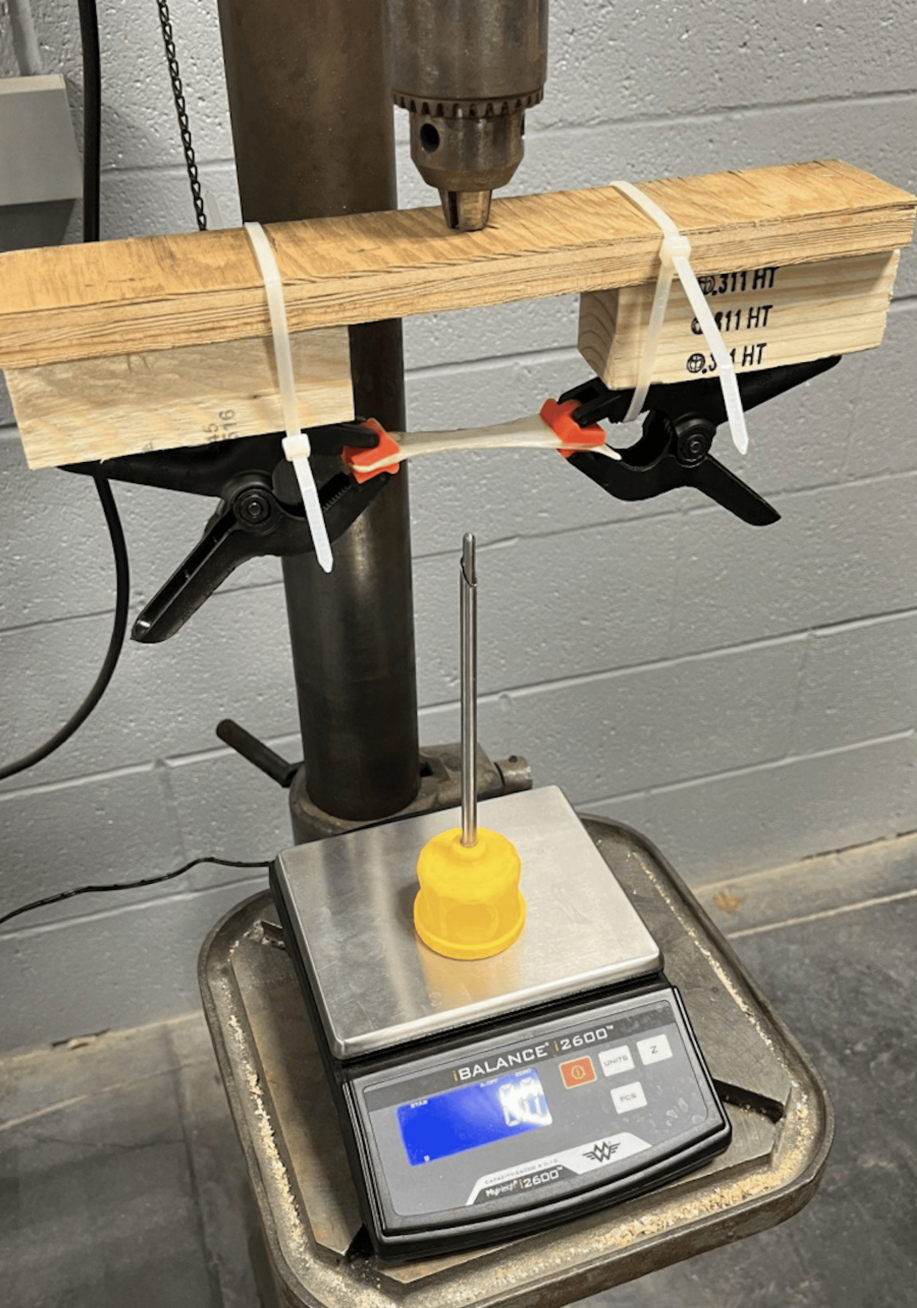
Mechanism used to test blade sharpness The system is positioned over a scale and zeroed. The sausage casing is placed between two clamps, and the mechanism exerts a force against the tip of the system. Image credits: authors of this article.

After determining which spring to use, the trocars were tested to see how much force was needed to pierce through a material similar to the peritoneum. Sausage casing was chosen to represent the peritoneum. This test was done with both the trocar designed by the team with the selected spring and the Versastep trocar. The trocar was placed upside down on the scale, and the scale was zeroed. A strip of sausage casing approximately one inch by one inch square and flattened to approximately 0.5 millimeters thick, simulating a single tissue layer, was prepared. The prepared casing was held securely above the scale using two clamps (Figure 2).

The average force required to break through the sausage casing was comparable for both trocars, around 2 and 2.98 N for the Verastrep and our prototype, respectively. 

Retraction speed testing

The final test measured the retraction speed (in seconds) of the dull shaft inside the sharpened pipe. This was done to ensure maximum safety for the organs inside the peritoneal cavity. A benchmark for the retraction time of the blade was set at less than 0.5 seconds in all different mediums. The trocar was tested in four different mediums: air, water (representing the fluid inside the peritoneal cavity), water mixed with honey (representing the fluid between the peritoneal layers), and water mixed with corn syrup (representing blood).

The average retraction speeds for air, water, water with honey, and water with corn syrup were 0.188, 0.176, 0.216, and 0.22 s, respectively.

The team concluded that the viscosities had little effect on the trocar system’s retraction speed. All testing indicated that the speed was below the mandated 0.5-s minimum.

## Discussion

Hydrocephalus remains a complex condition that requires innovative treatment approaches. Historical challenges have driven advancements in surgical techniques and the development of medical devices [5,6].

Hydrocephalus has a prevalence of 88/100,000 [95% CI: 72, 107] in pediatrics, 11/100,000 [95% CI: 5, 25] in adults, and 175/100,000 [95% CI: 67, 458] in older people. At birth, the incidence of hydrocephalus is 81/100,000 [95% CI: 69, 96] [1].

He et al. presented the results of a meta-analysis assessing the possible benefits of a laparoscopic-assisted insertion of the distal catheter [7]. They found that in the laparoscopy group, the rate of distal shunt failure was lower (OR: 0.41; 95% CI: 0.25, 0.67; p = 0.0003), operative time was shorter (mean difference [MD]: −12.84; 95% CI: −20.68, −5.00; p = 0.001), and blood loss was less (MD: −9.93; 95% CI: −17.56, −2.31; p = 0.01), demonstrating its benefits [7].

To date, laparoscopic insertion requires a general surgery team. In some reports, this involvement also allowed for better catheter positioning while decreasing operative time, length of stay, total revisions, and distal revision rates [8,9].

Ochalski et al. presented their technical report on using a minimal-access approach for distal catheter placement [10]. In their experience, they used a Veress needle and peel-apart percutaneous introducer. By using this technique, they avoid the need for an extra surgical team, but the method still relies on intraperitoneal insufflation and the creation of an additional access site [10]. Among other objectives, our prototype has been developed to surpass the need for an additional surgical team to insert the distal catheter by using a single-site, single-user feature while still ensuring patient safety. The results of the various safety and efficacy tests performed on the prototype reflect these objectives. 

The risk of laceration or perforation of intraperitoneal organs exists during laparoscopic trocar insertion [11]. It is hypothesized that the occurrence of internal organ injury during the insertion of laparoscopic trocars is due to the control and the force exerted by the surgeon [12].

Prototypes for distal peritoneal catheter placement have been previously presented. Wang et al. presented a trocar designed explicitly for the distal insertion of the catheter into the peritoneum [13]. It comprises two parts: a steel sheath and a steel core needle. The sheath had a gap in the lateral wall to allow the catheter to remain in place after introduction while the sheath was removed, similar to our prototype [13]. However, this prototype does not address the need for additional trocar insertion for the introduction of a laparoscope for direct visualization of the catheter placement [13,14].

**Figure 3:**
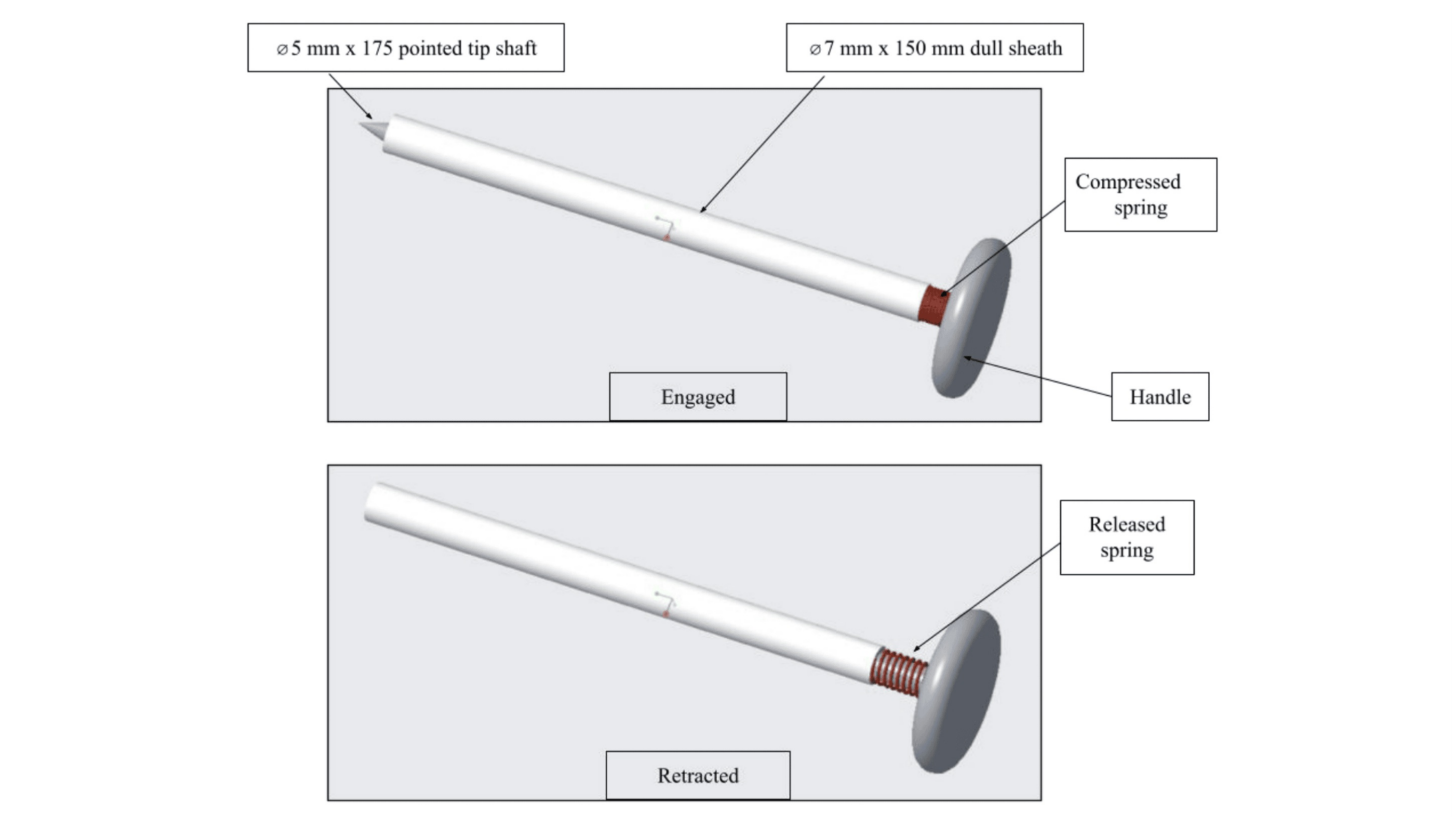
Initial prototype demonstrating the concept of using a spring under the handle that will provide retractability of the sharp shaft after insertion of the system Image credits: authors of this article.

One of the most significant features of our prototype is the addition of a sharp trocar shaft and a spring under the handle, with the capacity to retract after perforating the peritoneum to decrease the risk of organ injury (Figure 3).

When force is applied over the handle, with a tested force of 2.98 N, the sharp-tipped shaft is exposed to perforate the different abdominal layers. Once the peritoneum is accessed, the tip is retracted in less than 0.22 s, independently of surrounding fluids. The most distal portion of the system is the dull tip of the sheath, which protects internal organs from injury. By adding this capability, we also expect to avoid the need for peritoneal insufflation.

This device differs significantly from other laparoscopic trocars on the market, which rely only on the amount of force used during the system’s blind introduction, leaving a sharp tip visible even after accessing the cavity. The retractability and speed of retraction of the blade were comparable to those of the Versastep trocar. Different medium viscosities did not alter the retraction velocity, indicating relative safety while advancing through the abdominal wall layers.

Among the benefits of a single-use disposable device is the assurance of blade sharpness that is not always found in other devices after repeated use. This new system is being conceptualized, designed, and tested preliminarily. Therefore, in vivo clinical applications are still under investigation, and the potential benefits are purely speculative.

We encountered limitations. This is an initial prototyping, bench-only testing, and no in vivo validation has been completed in this phase. Therefore, this is a one prototype testing and is a small sample size with an untested clinical applicability.

## Conclusions

The trocar prototype showed functional reliability, low component-failure risk, and satisfactory sharpness after multiple laboratory tests. Because it is intended for single operator use, this self-retractable, disposable trocar system eliminates the need for assistance from a general surgery team during ventriculoperitoneal shunt placement. The safety features and innovative technology incorporated into the design eliminate the need for multiple trocar port sites as well as the need for intra-abdominal insufflation when placing the peritoneal portion of the catheter. This novel trocar system provides a safe, cost-effective alternative to more traditional methods of shunt placement. In light of these findings, further testing on animal models is warranted to demonstrate the in vivo benefits of this novel system.
